# Decoding Food Labels: A Study on the Knowledge, Attitudes, and Practices of Young Medical Students in Chennai

**DOI:** 10.7759/cureus.82657

**Published:** 2025-04-20

**Authors:** Arunkumar M, Elizabeth Varakumari, Ilamilaval Thozhanenjan, Vijayakarthikeyan M, Sujitha Pandian, Ashni Bhandari, Angeline Grace

**Affiliations:** 1 Community Medicine, Panimalar Medical College Hospital and Research Institute, Chennai, IND; 2 Community Medicine, Sri Venkateswaraa Medical College Hospital and Research Institute, Chennai, IND; 3 Community Medicine, Sree Balaji Medical College and Hospital, Chennai, IND; 4 Community Medicine, Vinayaka Mission's Kirupananda Variyar Medical College and Hospital, Vinayaka Mission’s Research Foundation (Deemed to Be University), Salem, IND

**Keywords:** awareness, food products, healthy diet, labels, nutritive value

## Abstract

Background

Food package labels play a crucial role in promoting healthy dietary choices by providing essential nutritional information. Medical students, as future healthcare professionals, must be well-informed about food labeling to guide patients in making healthier choices. This study aimed to assess the knowledge, attitude, and practices (KAP) regarding food package labels among Bachelor of Medicine and Bachelor of Surgery (MBBS) students in Chennai, South India.

Methods

A cross-sectional study was conducted among 425 undergraduate medical students. Participants were selected by their year of study using a stratified random sampling method. Data were collected using a structured, pre-tested questionnaire covering socio-demographics and KAP related to food package labels. Statistical analysis was performed using Statistical Package for Social Sciences (SPSS) version 22 (IBM Corp., Armonk, NY, USA), employing descriptive statistics, Chi-square test, and Kendall’s tau correlation to determine associations between variables.

Results

About 57% of the participants were females. A majority (96.9%) acknowledged the presence of food labels, and 86.1% understood their purpose. However, only 27.5% reported reading food labels regularly. While 65.2% demonstrated good knowledge, only 54.4% exhibited poor attitudes toward food labels despite recognizing their importance and 53% actively applied this knowledge in purchasing decisions. Female students had better knowledge (66.1% vs. 63.9%, p=0.02), but a more negative attitude compared to males (58.7% vs. 48.6%, p=0.01). Kendall’s tau correlation showed a significant positive relationship between KAP scores.

Conclusion

The findings of this study reveal a significant gap between knowledge and actual utilization of food package labels among MBBS students in Chennai. A relatively low percentage of students reported regularly reading labels or incorporating nutritional information into their purchasing decisions, indicating a disconnect between awareness and application. Furthermore, while female students showed a higher level of knowledge compared to their male counterparts, they also exhibited a more negative attitude toward the importance and utility of food labels. This knowledge-practice gap suggests that mere awareness is insufficient to influence health-conscious behavior and that underlying perceptions and motivations play a critical role in translating knowledge into practice.

## Introduction

It has been determined that food labels are a crucial public health tool for encouraging a balanced diet. Consumers can compare the nutritional values of similar food products and make informed, healthful food choices by using the information on food labels, which helps them better comprehend the nutritional value of food [1]. Customers are demanding more nutritional information as they become more conscious of the link between diet and illness. As a result, food labels help those on special diets (such as those with diabetes or high blood cholesterol) choose foods that are appropriate for their medical conditions [2]. Numerous significant diet-related illnesses and issues affect the general public, including poor nutrition, obesity, diabetes, cancer, hypertension, osteoporosis, and cardiovascular disease. According to the World Health Organization (WHO), dietary factors are responsible for about 30% of cancer cases in developed nations [3]. Customers believe they are informed about using food labels. The sections of the label that were read the most were those about calories, fat, sugar, salt, and fiber content. When making food purchases, they also consider serving size, ingredient lists, percentage daily values, pricing, brands, and health and nutritional claims. Many customers choose to use food labels rather than depending solely on their own expertise because they feel confident in their ability to read labels [4].

It is critical to evaluate consumers' attitudes toward choosing healthier foods as well as their degree of nutrition knowledge on dietary sugars, fats, and cholesterol. An analysis of consumer research on food labeling examined whether consumers utilize or read food labels when purchasing prepackaged foods [5]. Additionally, it has been noted that there was a positive correlation between the educational level of customers and their attitudes and knowledge. Higher-educated people are typically more open to learning about diet and health. Some studies have indicated that food label use decreases with age [5]. Studies on consumers' awareness of the nutritional aspects of using food labels in our nation are scarce. Although consumers knew how important it was to read food labels, it was clear that the most important information they focused on was the manufacturing date, expiration date, and contents of the package [6]. This may be explained by the fact that the majority of foods produced or repackaged in the area have labels that are devoid of many of the nutrition facts, serving sizes, unique characteristics, health claims, and health warnings that are mandated by Indian standards [7]. Considering the significant role of consumers’ awareness of food labels in making healthy food choices, it was, therefore, important to enlighten our community about the importance of reading food labels.

Among the consumers, medical students were considered a potential group for this study due to their critical role as future healthcare professionals. MBBS students will soon become doctors who advise patients on health and nutrition, and understanding food labels is fundamental in promoting healthy dietary choices [8]. Food labels provide key information on nutritional content, serving sizes, ingredients, and potential allergens, which are crucial for making informed decisions that can prevent and manage lifestyle-related diseases such as obesity, diabetes, and cardiovascular conditions. Assessing MBBS students' knowledge and attitudes toward food labels will highlight gaps in their awareness, enabling the development of targeted educational interventions to enhance their competency in this area [9]. Additionally, examining their personal practices regarding food labels will provide insights into how well they apply nutritional knowledge in their own lives, which can influence how effectively they counsel patients [10]. In the long term, improving MBBS students' knowledge and attitudes regarding food labels can contribute to more informed public health advice and promote healthier behaviors in the general population.

Hence, this study was conducted among undergraduate medical students in Chennai, South India, to assess consumers' Knowledge, Attitudes, and Practices (KAP) regarding food labels and to determine the association between KAP and the gender of the participants.

## Materials and methods

Study design

This study employed an analytical cross-sectional design to assess the KAP of undergraduate medical students regarding food package labels. A cross-sectional study was chosen as it allows for the collection of data at a single point in time, providing insights into the current state of awareness and behavior related to food labeling among medical students. This approach is particularly effective for identifying gaps in knowledge and variations in practices among different demographic groups within the study population.

Study area and population

The study was conducted in Chennai, Tamil Nadu, among undergraduate medical students. Specifically, the study targeted Phase 3 MBBS Part 1 and Part 2 students, as they had already completed foundational courses in Physiology, Biochemistry, and Community Medicine, which include essential nutritional concepts. Participants were required to be at least 18 years old and willing to provide informed consent.

Sample size and sampling method

In a study done by Robert SD and Chandran A [5] in South India, about 46% of participants read the food labels. A sample size of 382 was calculated at a 95% confidence interval (CI), with a margin of error (l) of 5%, using the formula Z^2^pq/l^2^, where Z=1.96, the proportion who read the food labels in the reference study (p)=46, and q=1-p. Accounting for 10% non-response, the final sample size for the study was 425. A stratified random sampling method was employed to ensure adequate representation of students from different academic years. The entire population of eligible Phase 3 MBBS students was first divided into strata based on their academic year, Part 1 and Part 2. This ensured proportional representation from both cohorts, minimizing sampling bias. Within each stratum, a simple random sampling technique was applied using student roll numbers as the sampling frame. This method ensured that every student within each academic year had an equal probability of being selected, thus enhancing the representativeness and generalizability of the study findings.

Data collection tool

Data were collected using a structured, pre-tested questionnaire designed based on previously validated studies on food label awareness and usage. The questionnaire comprised four major sections: (a) socio-demographic information: age, gender, year of study, place of residence, and any type of physical activity such as walking, jogging, swimming, etc.; (b) knowledge assessment: questions related to the components of food labels, their interpretation, and the significance of various nutritional information; (c) attitude assessment: participants' perceptions regarding the importance of food labels, their willingness to rely on food labels for dietary choices, and their confidence in interpreting the information; and (d) practice assessment: the frequency of reading food labels, specific sections of the label reviewed, and the influence of food labels on purchasing decisions.

To quantify KAP levels among participants, a pre-defined scoring system was employed based on responses to the structured questionnaire. Participants scoring ≥70% of the maximum possible score were categorized as having good knowledge, while those scoring below 70% were categorized as having poor knowledge. The cumulative attitude score was then categorized using the median split method, where scores equal to or above the median were classified as having a good attitude, and those below as poor attitude. Total practice scores were then categorized as good practice (≥60% of maximum score) or poor practice (<60%). The questionnaire was pilot-tested among 30 students who were not part of the final study to assess the clarity, reliability, and validity of the questionnaire. Necessary modifications were made based on feedback before administering the questionnaire to the study participants. The cut-off points were based on commonly accepted thresholds in similar KAP studies and were also informed by the distribution of scores from the pilot testing phase, ensuring cultural and contextual relevance.

Data collection procedure

Data were collected through self-administered surveys distributed both in paper format and online via Google Forms. Participants were given the option to choose either of the option. Written informed consent was obtained from all participants before they completed the questionnaire and electronic consent was obtained from those who participated through google forms. Participation was voluntary, and anonymity was maintained to encourage honest responses. Data collection was conducted over a period of four weeks. 

Data entry and statistical analysis

The collected data were entered into Microsoft Excel (Microsoft Corp, Redmond, WA) and subsequently analyzed using Statistical Package for Social Sciences (SPSS) software version 22. Descriptive statistics, such as frequencies and percentages, were used to summarize categorical variables. The mean and standard deviation were calculated for continuous variables. For analytical statistics, the Chi-square test was used to assess associations between categorical variables. A p-value of less than 0.05 was considered statistically significant. Kendall’s tau correlation coefficient was used to find the linear relationship between KAP scores. This method was favored over Spearman’s correlation due to the existence of tied ranks in the data, which Kendall’s tau addresses more effectively, particularly in intermediate sample sizes and ordinal datasets.

Ethical considerations

Ethical approval was obtained from the Institutional Human Ethics Committee of Sree Balaji Medical College and Hospital before commencing the study (Approval no: 001/SBMCH/IHEC/2024/2214). Participants were informed about the study’s objectives, procedures, and their right to withdraw at any stage without any consequences. All data were kept confidential and used solely for research purposes.

## Results

This analytical cross-sectional study assessed the KAP of undergraduate medical students regarding food package labels. A total of 425 medical students participated in the study, and their responses regarding socio-demographic determinants, as well as their knowledge, attitude, and behavior scores related to the purchase of processed food, were analyzed.

Socio-demographic characteristics

The mean age of the participants was 19.8 years (SD=1.36). Out of 425 respondents, 242 (56.9%) were female, and 183 (43.1%) were male (Table 1). The majority of the participants were unmarried (93.6%), and about 54.6% belonged to families with a total monthly income exceeding Rs. 75,000. Additionally, 71.1% of students reported living in households with four to six members. Physical activity levels varied, with 38.2% engaging in less than 30 minutes of daily exercise, while 30.9% exercised between 30 minutes and one hour per day.

**Table 1:** Socio-demographic profile of study participants (n=425)

S.no	Variable	Frequency	Percentage
1.	Gender		
	Female	242	56.9
	Male	183	43.1
2.	Age (in years)		
	18-20	316	74.4
	21-23	109	25.6
3.	Total number of household members		
	1-3	91	21.4
	4-6	302	71.1
	>6	32	7.5
4.	Total monthly income of the family		
	Less than Rs.25,000	62	14.6
	Rs.25,000-50,0000	72	16.9
	Rs.51000-Rs.75000	59	13.9
	More than Rs.75000	232	54.6
5.	Time spent in Physical activity per day		
	Nil	75	17.7
	<30 min	163	38.3
	30 min-1 hour	131	30.8
	>1 hour	56	13.2

Knowledge of food package labels

Among the participants, 96.9% acknowledged that food items come with package labels, and 86.1% understood their purpose (Table 2). The majority (81.4%) identified the back of the package as the most common location for labels. Furthermore, 81.2% recognized that consuming packaged processed foods could contribute to disease development. Regarding specific information included in food package labels, 87.8% were aware of manufacturing and expiry dates, 80.5% understood nutritional information, and 66.6% reported knowledge of preparation instructions.

**Table 2:** Knowledge of the participants on food package labels

S.no	Variables	Categories	Frequency	Percent
1.	Food items come with food package labeling	No	13	3.1
		Yes	412	96.9
2.	Understanding the purpose of food package labeling	No	59	13.9
		Yes	366	86.1
3.	Location of food package labels on the food items	Back side	346	81.4
		Front side	45	10.6
		Sides	24	5.6
		Do not know	10	2.4
4.	Use of packed processed foods leads to any disease	No	42	9.9
		Yes	345	81.2
		Do not know	38	8.9
5.	Information in the food package labels (multiple responses allowed)	Nutritional information	342	80.5
		Company and price details	38	8.9
		Manufacturing and expiry date	373	87.8
		How to use/prepare the food item	283	66.6

Attitude and perception toward food package labels

**Figure 1:**
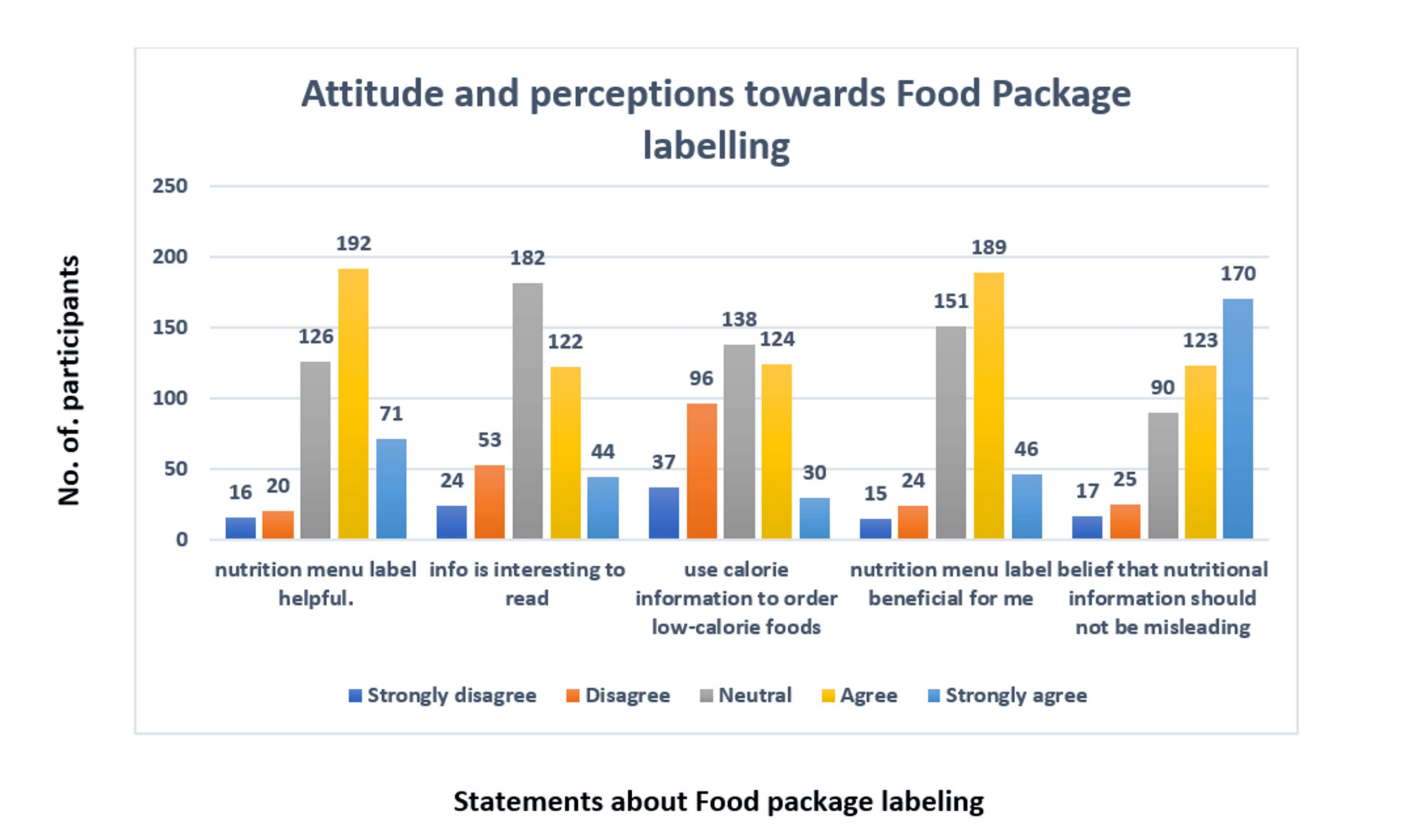
Attitude and perception toward food package labels

The majority of respondents agreed that nutrition labels were helpful in making informed dietary choices (Figure 1). Approximately 40% strongly agreed that nutritional information on food labels was not misleading. However, a discrepancy between perceived usefulness and actual reliance on food labels was observed, highlighting the need for better integration of nutritional knowledge into practice.

Practices related to food package labels

The practices on food package labels are summarized in Table 3. Regarding food label usage, only 27.5% of participants reported reading food labels regularly. While 63.1% of students used labels to choose food products, 73.4% avoided purchasing items that lacked labels. The most commonly checked sections included serving size (38.4%), total calories/energy (36%), and trans fat content (33.6%). However, only 34.1% of students calculated their calorie intake based on food label information.

**Table 3:** Practices on food package labels

S.no	Variables	Categories	Frequency	Percent
1.	Frequency of buying packed/processed food items	Daily	56	13.2
		Sometimes in a week	274	64.5
		Rarely	84	19.8
		Never	11	2.6
2.	Frequency of reading food package labels	Regularly	117	27.5
		Sometimes	275	64.7
		Never	33	7.8
3.	Choosing the product to buy based on the information provided in the food package label	No	157	36.9
		Yes	268	63.1
4.	Avoid buying a food product if there is no food package label	No	113	26.6
		Yes	312	73.4
5.	Calculate your calorie intake based on the information given in the food label	No	280	65.9
		Yes	145	34.1

Overall knowledge, attitude, and practice scores

**Figure 2:**
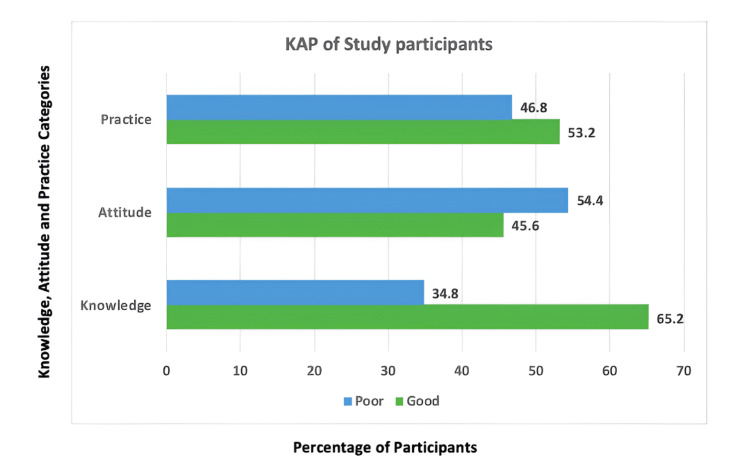
KAP of the study participants on food package labels KAP: knowledge, attitude, and practice

While 65.2% of students demonstrated good knowledge of food package labels, only 53% practiced reading labels while purchasing food products (Figure 2). Additionally, 54.4% of respondents exhibited poor attitudes toward food label usage, despite acknowledging their importance.

Association between knowledge, attitude, and practice and gender

The association between KAP and the gender of the participants is depicted in Table 4. Knowledge regarding food labels was higher among female students (66.1%) compared to males (63.9%), with a statistically significant association (p=0.02). However, a more negative attitude toward food labels was observed among female participants (58.7%) than males (48.6%) (p=0.01). No statistically significant association was found between gender and practice (p=0.06).

**Table 4:** Association of KAP with gender ^*^P-value is significant if <0.05 KAP: knowledge, attitude, and practice

S.no	Variable		Gender				P-value
			Female		Male		
			N	%	N	%	
1.	Knowledge	Good	160	66.1%	117	63.9%	0.02*
		Poor	82	33.9%	66	36.1%	
2.	Attitude	Negative	142	58.7%	89	48.6%	0.01*
		Positive	100	41.3%	94	51.4%	
3.	Practice	Good	115	47.5%	111	60.7%	0.06
		Poor	127	52.5%	72	39.3%	

Correlation between knowledge, attitude, and practice scores

Kendall’s tau correlation coefficient analysis (Table 5) indicated a positive correlation between knowledge and attitude scores (p=0.004) and attitude and practice scores (p<0.001). This suggests that improved knowledge may lead to more positive attitudes, ultimately influencing better food label-related practices.

**Table 5:** Relationship between knowledge, attitude, and practice scores ^**^Correlation is significant at the 0.01 level (2-tailed)

Correlations					
			Knowledge score	Attitude score	Practice score
Kendall's tau_b	Knowledge score	Correlation coefficient	1.000	.113^**^	.059
		Sig. (2-tailed)	.	.004	.121
		N	425	425	425
	Attitude score	Correlation coefficient	.113^**^	1.000	.275^**^
		Sig. (2-tailed)	.004	.	.000
		N	425	425	425

## Discussion

This study provided a quantitative analysis of MBBS students' KAPs regarding food package labels by administering a structured, pre-validated questionnaire to a representative sample of undergraduate medical students. The data were collected through self-reported responses, focusing on key domains such as awareness of nutritional information, frequency of label usage, and interpretation of health claims. The responses were statistically analyzed using descriptive and inferential methods to identify trends, associations, and potential gaps in understanding. The findings suggest that while knowledge of food package labels is relatively high among medical students, gaps exist in translating this knowledge into actual practice. The study highlights the need for targeted educational interventions to improve awareness and encourage more consistent use of food labels among future healthcare professionals. The importance of food labeling has grown with the increased awareness of nutrition, lifestyle diseases, and consumer rights.

Healthcare professionals, particularly MBBS students, are expected to have a deep understanding of nutrition and food labels as they guide patients toward healthier lifestyle choices [8,10]. In recent years, the importance of reading and understanding food labels has gained prominence as a public health tool, especially in tackling lifestyle diseases such as obesity, diabetes, and hypertension. Our findings indicate that while most medical students recognize the importance of food labeling, there remain significant gaps in their actual knowledge and practical application. Although the majority of students demonstrated a sound understanding of the presence and purpose of food labels, this knowledge was not consistently translated into practical behaviors. This aligns with previous studies that have reported similar discrepancies between theoretical understanding and real-world application of nutritional knowledge [7,8,11].

Our results show that a majority of medical students are aware of the existence of food labels and their importance in making informed dietary choices. However, when asked about specific components of food labels, such as serving sizes, percentage daily values, and nutrient reference values, many participants demonstrated incomplete knowledge. Similar findings were reported in a study by Campos et al., where consumers, despite being familiar with food labels, often misinterpreted critical nutritional information. This systematic review covered studies done among participants from various geographical regions and different age groups. The lack of comprehensive knowledge among MBBS students is concerning, given their role as future healthcare providers [2]. A study conducted among MBBS students in India found that while most students were aware of food package labels, their understanding of specific components such as macronutrients, portion sizes, and recommended daily intake values was often incomplete [11]. Research among Health sciences students in Saudi Arabia revealed that only 50% of the surveyed students checked the food labels before buying food items [12]. One study conducted in Turkey highlighted the lack of detailed knowledge about food allergens among restaurant personnel. Despite being a critical aspect of food labeling, fewer than half of the participants surveyed could identify common allergens such as gluten, dairy, or nuts on food labels [13]. Similar studies on food allergy and allergens are needed to be done among medical students to equip their knowledge. 

As emphasized by Sugimoto et al., doctors play a key role in educating patients about healthy dietary habits, and inadequate knowledge about food labeling can hinder their ability to provide effective nutritional counseling [8]. A study conducted by Miller and Cassady (2015) also found that while young adults had a positive perception of food labels, their ability to correctly interpret complex nutritional information such as values of macronutrients, minerals, and vitamins was limited [14]. Another study by Cowburn and Stockley (2005) concluded that while consumers frequently use food labels, comprehension levels are often low, particularly regarding macronutrient and calorie calculations [15]. Most participants in our study exhibited a positive attitude toward the importance of food labels. They acknowledged that reading food labels helps in making healthier food choices and agreed that food labeling regulations should be stringent to ensure consumer safety. However, despite this positive attitude, there was a noticeable gap between their beliefs and their actual practices. A possible explanation for this attitude-practice gap could be the limited emphasis placed on nutrition education in the medical curriculum. Coleman et al. have previously highlighted that while medical students recognize the significance of nutrition in disease prevention, their formal training in this area remains inadequate [9]. A study from a medical college in Saudi Arabia found that most MBBS students acknowledged the importance of food labels in promoting healthier eating habits. However, only a small percentage considered this knowledge essential for their future medical practice. This discrepancy suggests that while students may value food labeling on a personal level, they do not fully recognize its relevance in patient care [16]. Research from a university in South Africa showed that MBBS students were generally positive about using food labels in their personal lives, but they were less confident in counseling patients about nutrition. This lack of confidence was attributed to insufficient training in nutrition and food label interpretation during their medical studies [17]. A study from Brazil highlighted that MBBS students largely viewed food labeling as an important tool for public health but believed that government regulation was insufficient [18]. 

When it came to actual practices, our study found that only a moderate proportion of students consistently read food labels while purchasing packaged foods. The most frequently checked sections of food labels were expiry dates, ingredient lists, and calorie content. Other critical components, such as trans fats, sodium content, and serving sizes, were often overlooked. This is consistent with findings by Vijaykumar et al., who reported that supermarket shoppers in Singapore predominantly focus on basic information like expiration dates rather than detailed nutritional data [6]. One of the significant barriers to regular label usage among students was time constraints and difficulty in understanding technical terms. Many respondents reported that food labels often contain complex scientific terminology, making it difficult for the average consumer to interpret them accurately. This highlights the need for better consumer education and simplified labeling formats to enhance comprehension. A study by Grunert and Wills (2007) noted that while many European consumers claim to use food labels, their ability to make meaningful health decisions based on this information remains inconsistent [19]. This suggests that despite increased awareness, practical application still requires further improvement.

Several studies conducted in India and globally have explored consumer awareness and usage of food labels. Vemula et al. reported that urban consumers in India exhibit moderate levels of food label awareness but often lack an in-depth understanding of nutrition facts [7]. Similarly, research by Gandhi et al. emphasized the necessity of integrating nutrition education into the medical curriculum to improve knowledge and awareness among future doctors. The findings of this study underscore the urgent need to strengthen nutrition education within medical training programs [10]. A study in India found that while a majority of students reported occasionally reading food labels, only 30% did so consistently. The primary reasons for not using food labels included time constraints, difficulty in understanding the information, and a lack of perceived need, especially when purchasing familiar products [11]. Research from a medical college in Indonesia found that students who regularly used food labels made healthier dietary choices, opting for foods lower in sugars, unhealthy fats, and sodium. However, only a small fraction of students reported using food labels to manage their own health, such as controlling weight or preventing diseases like hypertension or diabetes [20]. A study from a medical university in Malaysia revealed that while students who read food labels were more likely to plan balanced meals, most still did not prioritize nutritional information in their meal planning. Instead, factors such as taste, convenience, and price often overshadowed the nutritional benefits when making food choices [21].

Given the rising burden of lifestyle diseases such as obesity, diabetes, and cardiovascular diseases, equipping future doctors with robust knowledge about food labels is crucial in promoting healthier dietary habits among the general population. Medical curricula should incorporate more extensive training on food labeling, including practical workshops on interpreting nutritional information. Conducting awareness programs for medical students about the importance of food labels could enhance their knowledge and encourage better practices. Hands-on workshops where students practice reading and interpreting food labels could help solidify their understanding and make this a more practical part of their medical education. MBBS students should be trained in counseling patients about nutrition, including how to use food labels to make healthier choices. This training should emphasize the importance of food labeling in managing lifestyle diseases. Regulatory bodies should consider revising food labels to make them more user-friendly and easily interpretable. Students should be encouraged to actively practice reading and analyzing food labels in their daily lives, thereby reinforcing their theoretical knowledge with real-world application.

Limitations of the study

While this study provides valuable insights into medical students' KAP on food package labels, certain limitations must be acknowledged. The study included a limited population and the results may not reflect broader trends. A larger and more diverse study population would improve the study's external validity. Data collection relied on self-reported responses and there is a risk of social desirability bias. Also, a cross-sectional study design captures only a single point in time. Longitudinal studies could provide a better understanding of trends over time. 

## Conclusions

The KAP of MBBS students regarding food package labels reflects a critical area that requires improvement. While medical students generally recognize the significance of food labeling in making informed dietary choices, there are notable gaps in their understanding and practical application. Many students may be aware of the existence of nutrition labels but lack the necessary skills to interpret them effectively. This limitation not only affects their personal dietary habits but also reduces their ability to guide patients toward healthier nutrition choices. Given the growing prevalence of lifestyle-related diseases such as obesity, diabetes, and cardiovascular conditions, the ability to analyze and apply food label information is becoming increasingly essential in medical practice. Addressing these gaps through targeted education and training will help ensure that future healthcare professionals are well-equipped to incorporate nutrition counseling into their practice.

As the role of nutrition in healthcare continues to grow, the ability of MBBS students to effectively utilize food labels will be crucial in addressing lifestyle-related diseases and promoting public health. Well-informed doctors can significantly influence patient behavior by providing evidence-based dietary guidance tailored to individual needs. Additionally, by fostering a culture of nutrition awareness within the medical community, healthcare professionals can serve as role models, encouraging patients and the public to make informed food choices. A comprehensive approach to nutrition education, one that includes theoretical knowledge, practical training, and public health advocacy, will not only enhance medical students’ competencies but also contribute to long-term improvements in public health outcomes. Therefore, prioritizing food label literacy in medical education is a necessary step toward creating a healthcare system that emphasizes prevention alongside treatment.

## Appendices

                                         Survey on Consumer Knowledge, Attitude, and Practice Regarding Food Package Labels

Part I - Demographic Details

1. Age (years):

2. Gender

a)     Male

b)     Female

3. Total number of household members:

a)     1-3

b)     4-6

c)     >6

4. Marital Status:

a)     Married

b)     Unmarried

c)     Widowed

d)     Separated

e)     Other:

5. Total monthly income of the family:

a)     Less than Rs.25000

b)     Rs.25,000-50,0000

c)     Rs.51000-Rs.75000

d)     More than Rs.75000

6.  Time spent in physical activity (average time per day):

a) nil

b) less than 30 minutes

c) 30 minutes to 1 hour

d) more than 1 hour 

Part II - Knowledge Regarding Food Package Labeling

1. Do you know food items come with food package labeling? 

a)     Yes

b)     No

2. Do you know what purpose these food package labeling serve to consumers? 

a)     Yes

b)     No

3. Where are these food package labels usually present on the food items? 

a)     Front Side

b)     Back Side

c)     Sides

d)     Don't Know

4. Can you recall what information these food package labels contain? (multiple responses)

a)     Related to manufacturing and Expiry date

b)     Related to Nutritional information of the product

c)     Related to the Direction of How to use/prepare the food item

d)     related to company name and price details

5. Do use of these packed processed foods lead to any disease?

a)     Yes

b)     No

c)     Don't know

6. Packed processed foods high in salt, sugar and fat can cause diseases related to (multiple responses)

a)     Heart

b)     lung

c)     liver

d)     Stomach

e)     Blood vessels

f)      All

g)     None

h)     Other:

Part III - Attitude and Perceptions Toward Food Package labeling

1. I found nutrition menu label helpful.

a)     Strongly disagree

b)     Disagree

c)     Neutral

d)     Agree

e)     Strongly agree

2. It is interesting for me to read the nutrition menu label always.

a)     Strongly disagree

b)     Disagree

c)     Neutral

d)     Agree

e)     Strongly agree

3. I would use calorie information to order low-calorie foods and drinks.

a)     Strongly disagree

b)     Disagree

c)     Neutral

d)     Agree

e)     Strongly agree

4. It is beneficial for me to read the nutrition menu label.

a)     Strongly disagree

b)     Disagree

c)     Neutral

d)     Agree

e)     Strongly agree

5. I trust the nutritional information provided on the nutrition menu label.

(1 - No, 5 - Full trust)

                                    1          2          3          4          5

6. The nutritional information provided affects my decision to purchase.

(1 - doesn't affect, 5 - fully affect)

                                    1          2          3          4          5

7. Which do you think is very important in a food label?

a)     Price

b)     Expiry date

c)     Ingredients

d)     Nutrition information

e)     Brand name

8. I believe nutritional information should not be misleading.

a)     Strongly disagree

b)     Disagree

c)     Neutral

d)     Agree

e)     Strongly agree

9. Nutritional information (carbohydrate, protein, fat) indicated by percentage is sufficient for me to know how many ingredients the food contains.

a)     Yes

b)     No

c)     Need additional information

Part IV - Practices on Food Package Labels

1. How often do you buy packed/processed food items?

a)     daily

b)     sometimes in a week

c)     rare

d)     never

2. Why do you buy packed foods? (multiple responses)

a)     Cheap

b)     easily available

c)     tasty

d)     ready to eat

e)     accessible

f)      not applicable

3. How often do you read food package labels?

e)     regularly

f)      sometimes

g)     never

4. How often do you notice/read the following items in the food package label?

(often/sometimes/rarely/never)

a. List of ingredients

b. Serving size

c. Health claim

d. Calories/energy

e. Calories from Fat

f. Total fat

g. Trans fat

h. Saturated fat

i. Cholesterol

j. Sodium/salt

k. Carbohydrate

l. Protein

m. Fiber

n. Sugar

o. Vitamins and minerals

5. Will you choose the product to buy based on the information provided in the food package label?

a)     Yes

b)     No

6. Will you avoid buying a food product if there is no food package label?

a)     Yes

b)     No

7. Do you calculate your calorie intake based on the information given in the food label?

a)     Yes

b)     No

                                                                                 Thank you for participating in the survey.
